# Abortion attitudes, religious and moral beliefs, and pastoral care among Protestant religious leaders in Georgia

**DOI:** 10.1371/journal.pone.0235971

**Published:** 2020-07-17

**Authors:** Jessica L. Dozier, Monique Hennink, Elizabeth Mosley, Subasri Narasimhan, Johanna Pringle, Lasha Clarke, John Blevins, Latishia James-Portis, Rob Keithan, Kelli Stidham Hall, Whitney S. Rice

**Affiliations:** 1 Department of Population, Family and Reproductive Health, Johns Hopkins Bloomberg School of Public Health, Baltimore, Maryland, United States of America; 2 The Center for Reproductive Health Research in the Southeast, Rollins School of Public Health, Emory University, Atlanta, Georgia, United States of America; 3 Hubert Department of Global Health, Rollins School of Public Health, Emory University, Atlanta, Georgia, United States of America; 4 Department of Behavioral, Social and Health Education Sciences, Rollins School of Public Health, Emory University, Atlanta, Georgia, United States of America; 5 Department of Epidemiology, Rollins School of Public Health, Emory University, Atlanta, Georgia, United States of America; 6 Graduate Division of Religion, Laney Graduate School of Arts and Sciences, Emory University, Atlanta, Georgia, United States of America; 7 Reproductive Justice Activist and Movement Chaplain, Atlanta, Georgia, United States of America; 8 All Souls Church Unitarian, Washington, D.C., United States of America; 9 Heilbrunn Department of Population & Family Health, Mailman School of Public Health, Columbia University, New York, NY, United States of America; Coventry University, UNITED KINGDOM

## Abstract

**Objective:**

The purpose of this study is to explore Protestant religious leaders’ attitudes towards abortion and their strategies for pastoral care in Georgia, USA. Religious leaders may play an important role in providing sexual and reproductive health pastoral care given a long history of supporting healing and health promotion.

**Methods:**

We conducted 20 in-depth interviews with Mainline and Black Protestant religious leaders on their attitudes toward abortion and how they provide pastoral care for abortion. The study was conducted in a county with relatively higher rates of abortion, lower access to sexual and reproductive health services, higher religiosity, and greater denominational diversity compared to other counties in the state. Interviews were audio-recorded, transcribed verbatim, and analyzed by thematic analysis.

**Results:**

Religious leaders’ attitudes towards abortion fell on a spectrum from “pro-life” to “pro-choice”. However, most participants expressed attitudes in the middle of this spectrum and described more nuanced, complex, and sometimes contradictory views. Differences in abortion attitudes stemmed from varying beliefs on when life begins and circumstances in which abortion may be morally acceptable. Religious leaders described their pastoral care on abortion as “journeying with” congregants by advising them to make well-informed decisions irrespective of the religious leader’s own attitudes. However, many religious leaders described a lack of preparation and training to have these conversations. Leaders emphasized not condoning abortion, yet being willing to emotionally support women because spiritual leaders are compelled to love and provide pastoral care. Paradoxically, all leaders emphasized the importance of empathy and compassion for people who have unplanned pregnancies, yet only leaders whose attitudes were “pro-choice” or in the middle of the spectrum expressed an obligation to confront stigmatizing attitudes and behaviors towards people who experience abortion. Additionally, many leaders offer misinformation about abortion when offering pastoral care.

**Conclusion:**

These findings contribute to limited empirical evidence on pastoral care for abortion. We found religious leaders hold diverse attitudes and beliefs about abortion, rooted in Christian scripture and doctrine that inform advice and recommendations to congregants. While religious leaders may have formal training on pastoral care in general or theological education on the ethical issues related to abortion, they struggle to integrate their knowledge and training across these two areas. Still, leaders could be potentially important resources for empathy, compassion, and affirmation of agency in abortion decision-making, particularly in the Southern United States.

## Introduction

Abortion is a common experience for U.S. women of reproductive age—approximately 1 in 4 will have an abortion by age 45 [1]. In the United States, 13.5 abortions per 1,000 women of reproductive age were reported in 2017. While national and regional abortion rates have been declining, rates in the state of Georgia have increased in recent years [2]. The rate of abortion in Georgia was 16.9 abortions/1,000 women aged 15–44 in 2017 [2].

Despite abortion being common, most people in Georgia face systemic and socio-cultural barriers limiting access to abortion services. Attitudes that condemn abortion emerge in policy, systems, and at the community-level [3]. Manifestations of abortion stigma [3–6] may influence people’s ability to exercise reproductive autonomy [7, 8]. Researchers suggest “abortion stigma confounds a woman’s decision to terminate a pregnancy due to worries about judgment, isolation, self-judgment, and community condemnation” [9]. In 2014, only 4% of counties had clinics that provided abortion, leaving 58% of women in Georgia without a clinic in their county. Restrictive state legislation threatens to impose additional barriers to abortion access. For example, in 2019, Georgia legislators introduced HB 481, a bill that sought to outlaw abortion once a fetal heartbeat is detected [10]. This bill aimed to restrict abortion as early as 6 weeks gestation, before many people know they are pregnant [11]—one of the strictest abortion bans in the nation [12]. A federal judge granted an injunction in early October 2019, which blocked the law while it is argued in court [13].

More restrictive policies leave the most vulnerable, economically disadvantaged, and socially isolated people with few choices but to carry pregnancies they feel they are unable to support to term; however, those who carry pregnancies also face systemic barriers including limited access to perinatal care. Georgia has one of the highest maternal mortality rates in the United States [14], yet access to obstetric services is limited by a declining obstetrician/gynecologist workforce, especially in rural areas [15]. Moreover, half of all counties in Georgia are without a single obstetrician/gynecologist or hospital where women can give birth or access basic services [16].

Religious leaders are pivotal in their faith communities [17] and may be influential in shaping attitudes towards sexual and reproductive health (SRH), norms, and behaviors at the individual, family and community levels [8, 17, 18]; however, religious doctrine and beliefs may come in direct conflict with public health recommendations regarding abortion and contraception [19]. Previous research [4] has found links between religiosity and experiences of abortion stigma and it is “often the religious voices that oppose sexual and reproductive rights that have been the most visible in the media and most influential in policy debates” [17]. In a landmark 2014 case, *Burwell v Hobby Lobby Stores*, *Inc*., the Supreme Court ruled that the Religious Freedom Restoration Act (RFRA) of 1993 allows a for-profit company to deny its employees coverage for contraception through the employer-based health plan because of the religious objections of the Hobby Lobby owners [20]. Conversely, Mainline Protestant religious leaders have historically played a role in shaping SRH policy, specifically with advocacy efforts for increased access to contraception and abortion during the twentieth century [17].

While many religions are perceived to condemn abortion [21], religiously affiliated women do have abortions—the majority (62%) of women who obtained an abortion in 2008 and 2014 claimed a religious affiliation [22]. Of these women, 17% identified an affiliation with a Mainline Protestant denomination [22]. This percentage is higher than that found in the American population as a whole; in 2010, Mainline Protestants only represented 7.2% of the United States population [23].

Religious leaders may play an important role in providing SRH-related pastoral care and resources given that “religions have a venerable tradition supporting healing, health care, disease prevention, and health promotion [and a] commitment to the most marginalized, the most vulnerable, and the most likely to be excluded” [17]. Still, little is known about the pastoral care practices of religious leaders as they relate to abortion, especially in the southern states of the United States and there are few resources in pastoral theology that address abortion. In fact, a review of publications from Mainline Protestant publishing houses over the last two decades identified only two books published on pastoral care and abortion [24, 25]. A keyword review of the American Theological Library Association (ATLA) database identified no peer-reviewed articles exploring abortion published in pastoral theology journals over the last twenty years. Given their potential reach and influence, there is a need to understand the religious and moral views that shape religious leaders’ attitudes toward abortion, and their pastoral care practices, particularly in Georgia, a state with high religious influence [26] and gaps in reproductive healthcare [15, 16, 27]. This study aims to explore Mainline and Black Protestant religious leaders’ attitudes towards abortion and how they provide pastoral care regarding abortion.

## Materials and methods

### Participant recruitment

Participants were recruited from two religious traditions based on categorization developed by Steensland and colleagues [28]. Mainline Protestantism is a branch of Protestantism that consists of denominations that are generally considered theologically liberal and moderate (e.g. Presbyterian Church (USA), the United Methodist Church, Episcopal Church, and the United Church of Christ). Black Protestantism (also known as “the Black Church”) is theologically and structurally similar to white evangelical denominations, “but also emphasizes social justice and community activism” [29]. According to the Association of Religious Data Archives, Black Protestant denominations are generally economically liberal and socially conservative [29]. The tradition consists of seven major denominations, such as the African Methodist Episcopal Church, the Church of God in Christ, and the Progressive National Baptist Church [29].

For this study, 20 semi-structured, in-depth interviews were conducted with religious leaders serving in Mainline and Black Protestant churches in a county with urban and rural areas outside of Atlanta, Georgia. The study site was selected due to its higher abortion rates, lower SRH service access, a higher religious adherence, greater denominational diversity, and an abundance of Mainline Protestant and Black Protestant churches as compared to other similar counties in Georgia. Religious leaders were eligible to participate if they were currently serving in a Mainline Protestant or Black Protestant church as a clergy member or lay leader (i.e. a non-ordained member of a Christian church [29]) for at least 6 months prior to the interview, were over 18 years old, and spoke English.

Purposive sampling was used to recruit a sample of religious leaders diverse in denomination and sociodemographic characteristics. Churches were identified using a publicly available list of churches by county published by The Association of Religious Data Archives. Names and contact information for the primary religious leader of these churches were obtained from church websites and social media. Lay leaders were recruited by social media messages and snowball sampling because their contact information was often not publicly available on church websites. Religious leaders were contacted up to five times by email, phone, and social media message using standard Institutional Review Board (IRB)-approved scripts. Leaders were also approached in person and recruited at centers of commerce (e.g. local strip malls) in the county using a standard recruitment script, screened for eligibility, and interviewed at a later date in a private location.

### Participant characteristics

Socio-demographic characteristics of study participants are presented in Table 1. The majority of participants recruited were men (80%) serving in senior pastoral roles (60%). Other religious titles reported included: pastor, associate pastor, first lady, minister, regional minister, youth minister, and lay leader. The average length of time served in the participant’s current role was 8.3 years (6 months to 40+ years). Age ranged from 28 to 72 years, with a mean age of 48. Sixty-five percent of participants had a graduate degree—most commonly a Masters of Divinity. Church membership size ranged from less than 50 to over 1000 people.

**Table 1:** Socio-demographic characteristics of religious leaders.

Characteristics		n (%)
**Race**		
	White	10 (50)
	Black	10 (50)
**Age**		
	18–34	4 (20)
	35–50	6 (30)
	51–69	9 (45)
	70+	1 (5)
**Marital Status**		
	Married	14 (70)
	Divorced	2 (10)
	Remarried	1 (5)
	Single (Never Married)	2 (10)
	Not reported	1 (5)
**Political affiliation**		
	Democrat	13 (65)
	Independent	4 (20)
	Republican	3 (15)
**Tradition**		
	Mainline Protestant	11 (55)
	Black Protestant	9 (45)
**Denomination**		
	United Methodist Church	5 (25)
	Evangelical Lutheran Church in America	1 (5)
	Black Protestant–Baptist Unspecified	3 (15)
	Christian Church (Disciples of Christ)	1 (5)
	Congregational Methodist Church	1 (5)
	Episcopal Church	1 (5)
	African Methodist Episcopal (AME)	2 (10)
	International Pentecostal Holiness Church	1 (5)
	National Baptist Convention USA	2 (10)
	National Missionary Baptist Convention Inc.	1 (5)
	Non-denominational	1 (5)
	Presbyterian Church USA	1 (5)

### Data collection

In-depth interviews were conducted between October 2018 and September 2019 using a semi-structured interview guide (See S1 Appendix). The interview guide included questions on participants’ views about unplanned pregnancy and abortion, pastoral care on these topics, and suggestions for discussions and programming around unintended pregnancy and abortion in faith settings. Some of the questions included, “*What are your personal views on abortion*?*”* and “*What advice would you give someone in your congregation considering abortion*?” Researchers probed for barriers and facilitators to providing pastoral care on these topics and specific resources and scripture religious leaders would rely on during these conversations. The interview guide was pilot-tested in four interviews with clergy members serving in Mainline and Black Protestant churches in metro-Atlanta and then refined for clarity. Iterative changes were made to the interview guide and to participant recruitment throughout the data collection period to explore new topics raised and to include participants with differing perspectives, as is usual in qualitative data collection.

Participants were consented verbally. They were read an IRB-approved consent script, invited to ask follow-up questions, and asked if they agreed to take part in the study. A short demographic survey was administered after the informed consent process. Interviews were conducted in private offices at the participant’s church, at Emory University, or another location (e.g. a private room in a coffee shop) and digitally audio-recorded. Interviews lasted between 45 and 90 minutes, and participants received a $50 gift card. The study was approved by the IRB at Emory University (IRB 00106069).

Five researchers collected the data. They were trained on qualitative research methods, the study protocol, and research ethics. All researchers were cisgender women of reproductive age: four multi-ethnic women and one white woman. They had varying religious backgrounds and level of experience within Christian churches. Throughout data collection, the researchers practiced reflexivity by journaling their own thoughts and impressions about the research topic or participant’s experiences. They also noted potential biases that may have influenced data collected. Researchers shared these reflexive notes in team debriefing sessions and discussed ways to minimize researcher influence on the data collection process. Debriefing sessions included discussions on how researcher’s social identities and preconceptions about the study issues may have influenced the data as well as strategies to minimize these influences. For example, reflexivity revealed emerging data on race and religion, whereby the team felt that matching the race of researchers and participants may provide a more enabling environment for a deeper, more nuanced data on participants views on race and abortion.

### Data analysis

Thematic analysis was used to describe Mainline and Black Protestant religious leaders’ attitudes towards abortion and how they provide pastoral care regarding abortion. Interviews were transcribed verbatim by a professional transcription company and de-identified by the research team. Data were managed and organized using Dedoose version 8.0.35, a software package for qualitative data analysis [30]. Data collection, coding, and analysis occurred simultaneously to assess meaning saturation, or a “richly textured understanding” of abortion attitudes and pastoral care practices [31]. Saturation was reached at 20 interviews when a diversity of views and denominations was achieved and no new themes were observed.

Data were read in detail and memoed in order to develop a codebook (See S2 Appendix). Codes were developed through inductive (emerging from the data) and deductive (pre-determined topics from the interview guide) strategies. An inter-coder agreement exercise was conducted prior to coding all data to ensure consistency in the coding process. Weekly team meetings were held to refine code definitions, resolve discrepancies in coding, and discuss reflexivity in data interpretation during the coding process. For example, researchers discussed how our underlying epistemologies, public health training, and differing views on scripture and doctrine, might influence interpretation of data during analysis.

Researchers explored data by codes (e.g., *abortion*, *attitudes & beliefs*, and *pastoral care)*, conducted structured comparisons of codes by sub-groups of participants (e.g., by sociopolitical attitudes, denomination, and gender). Patterns in the data were examined and sub-themes within codes were identified. Illustrative quotes were then selected for each sub-theme.

## Results

Results were organized around 1) abortion attitudes; 2) moral and religious beliefs; and 3) pastoral care.

### Abortion attitudes

When asked about their views on abortion, most participants noted affiliation with sociopolitical attitudes regarding abortion (e.g. “pro-life” and “pro-choice”). Differences among attitudes were observed in the participant’s understanding of when life begins, an affirmation of a women’s autonomy, and expression of the circumstances in which abortion may be morally acceptable. All participants identified at least one circumstance in which abortion may be the best decision for a pregnant person. Participants who identified their views as “pro-life” offered fewer moral exceptions for abortion, explaining that the circumstances of most unplanned pregnancies are surmountable, and therefore do not need to be resolved by abortion.

Nonetheless, the majority of participants expressed statements not readily fitting into a dichotomy of attitudes, but rather intermediate between “pro-life” and “pro-choice” in the so-called “gray area.” Attitudes in the “gray area” were nuanced, complex, and fell along a spectrum between “pro-life” and “pro-choice” attitudes. “Gray Area” attitudes were distinguished by an understanding that people have to make decisions on their own, yet “all life is sacred” and should be protected. In addition, participants with these “gray area” attitudes expressed tentativeness about taking a strong stance of “pro-life” or “pro-choice,” noting tensions between beliefs held in both categories and a desire to hold onto religious beliefs while acknowledging legal right to abortion. Illustrative quotes of the range of common attitudes are presented in Table 2.

**Table 2:** Illustrative quotes of abortion attitudes.

Common Themes	Illustrative Quotes
Pro-life	“Pro-life but not in a Republican religious right type of way … pro-life for the baby … for the mother, and for people when it comes to healthcare.”
	“I’m not for abortion … you don’t give a woman the right to make the decision whether or not to kill when she’s pregnant.”
Attitudes in the “gray area”	“I tend to be more conservative on this issue. I'm not blockading abortion clinics, but I'm not out waving the banner of pro-choice either. So I guess I'm somewhere in the middle. But I would hold to the sanctity of life, and share that with people, without a doubt.”
	“That's my tension. It's like I'm not for it at all, but (God) gives me free choice every day. So that's my dilemma. Now if you were to ask me what I vote for, pro-life, pro-choice, I'm definitely pro-life. But I still—that's the tension a little bit. All right? 'Cause it doesn't stop.”
	“[The church’s] decision probably has always been pro-choice. BUT we advocate life.”
Pro-choice	“I’m a firm believer that it’s a woman’s right to choose. It’s your body … and it’s your life. … I love adoption. I think that’s a great option, but it’s an option. … I don’t know every person’s personal story, what they’ve gone through, what they have to go through. So, I don’t know that it has anything to do with me, so why should I have any determination or even any philosophy or theology on it?”
	“We have a very conservative (denomination). For the most part, we'll use the word progressive, they're on the progressive scale. I mean, gay marriage–we changed our canons to allow for gay marriage. We're not against contraception. We are–we consider ourselves pro-choice. When we say it, we mean pro-family, pro-child, and life begins, and life doesn't end until a person dies, not by the hands of the state.”

#### “Pro-life”

There was no pattern among participants with “pro-life” attitudes by gender, tradition, denomination, or leadership role; participants included both men and women, senior pastors and a first lady and other lay leaders, and came from multiple denominations, including United Methodist Church, Congregational Methodist, National Missionary Baptist Convention, Inc., and a Non-denominational congregation. Those with “pro-life” attitudes felt abortion is “too common” and “ought to be a last resort” that is not rushed into or taken lightly given the gravity of its implications. They expressed perceptions that abortion is too often thought of as a first option and explained that they would encourage congregants to make well-informed, carefully considered decisions when faced with an unplanned pregnancy. Moreover, participants with “pro-life” attitudes explained that abortion is too often discussed in a “cold and sterile” medical manner. They explained that this perspective is limited because it presents abortion only as a solution to a medical problem, but detaches moral implications of ending the “potential for life.” Participants with “pro-life” attitudes explained it is impossible to honestly discuss abortion in only medical terms; morality must also be considered and negotiated. These participants emphasized the importance of sharing religious beliefs of life and scared worth when providing pastoral care for someone considering abortion.

Participants with “pro-life” attitudes acknowledged abortion as a legal option but explained they would only counsel women to consider this recourse in cases of risk to maternal life or in some cases of sexual assault. Some participants were less decided about the moral acceptability of abortion in cases of rape, stating tensions between the belief that something good might come out of the pregnancy and concerns for the mental health of the mother. One Mainline Protestant pastor raised concerns about abortion in cases of rape and expressed that it may be morally acceptable only when the woman has no shared “responsibility for [the pregnancy],” such as a woman being under the influence of alcohol versus being “attacked by an evil person.” The same participant explained that abortion is not a morally acceptable option for fetal anomaly because such anomalies are the result of “the sinful nature that we’re born into (due to original sin of Adam and Eve)”, and because God does not make mistakes, therefore all pregnancies should be carried to term.

#### Attitudes in the intermediate, “Gray Area”

Participants from several denominations such as United Methodist Church, Evangelical Lutheran Church in America, African Methodist Episcopal expressed attitudes intermediate between “pro-life” and “pro-choice.” These participants were tentative about making strong statements about what the ideal resolution of unplanned pregnancies should be and explained that they were not “qualified” to make such determinations for others. Several participants expressed the importance of individual autonomy to make abortion decisions and careful consideration of the context of an unplanned pregnancy in deciding on the ideal resolution of the pregnancy; however, most of the participants with attitudes in the “gray area” expressed that they would prefer abortions are less common. Participants cited tension between belief in the sanctity of life and respect for individual autonomy. One participant described this tension:

*“'So I don't believe that it's my right as a human being to tell a woman what they're going to do with their body because a woman and a child are literally inexorably linked as far as being in utero and in womb*. *And so I have no right to say to someone who's carrying a child that you can or cannot do this or that or the other because that child is your body and you have the right to see your body*. *But at the same time*, *there is also the potential for a life being carried inside of that body*. *And the part of me that values the sacredness of all life says*, *‘Oh*, *but look at the potential there*. *Look what good that future human being could do in the world*.*’ So I sort of stand at a very strange gray area and crossroads with abortion*.*”* (Senior Pastor, Mainline Protestant)

Some participants in the “Gray Area” cited that abortion may be the best decision for some people if having an abortion would alleviate potential suffering or in cases in which a mother is not able to care for herself or for a child. Another senior pastor explained his view on abortion,

*“My personal views on abortion is that I believe that in some cases*, *abortion could be the best option for that individual*, *if they come to that conclusion*, *such as poorly–development of a fetus that does not have*, *medically speaking*, *the chance for–productive or normal life outside of the womb*. *Okay*? *People make those decisions on their own*. *One of the more sensitive issues is having a healthy child*, *but if that child is the result of rape or incest*, *I don't believe that God cannot forgive anyone for any decision that possibly could be against his will*. *His will*, *of course*, *is that we have life*, *but I also hold to this belief that every*, *every*, *every–conceivable sin is forgivable by God*, *except for blasphemy …”* (Senior Pastor, Black Protestant)

#### “Pro-choice”

There was no identifiable pattern by gender, tradition, denomination, or leadership role among participants who identified themselves as “pro-choice.” These participants discussed tensions between the need for abortion and the need for women to exercise bodily autonomy. They felt pregnancy-related decision making should rest with a pregnant person and God, but they would try to guide people considering abortion to the best outcome for the mother and the baby. They emphasized that their pastoral care would consist of much listening and understanding. A senior pastor at an Episcopal church who identified as “pro-choice” explained that he could not make decisions about abortion for people because to do so would be “treading on a violation of the relationship between [them] and God.”

Most participants with “pro-choice” attitudes expressed that abortion is a psychologically difficult decision that they wished people did not have to go through but underscored that it may be the best option for some people. These participants explained that abortion might be the best decision if there is a risk to the life of the mother, in cases of rape or incest, and in cases of fetal anomaly. Conversely, some participants expressed that abortion should not be used as a primary means of birth control or contraception. A senior pastor at an Episcopal church expressed that abortion should not be allowed for sex-selection, although he did not believe this was a common occurrence. Several other participants acknowledged abortion as a legal option but emphasized the importance of supporting women and providing children the care they need so there are better alternatives to abortion.

### Religious and moral beliefs across the abortion attitude spectrum

#### All life has sacred worth

All participants expressed that their abortion attitudes are influenced by the understanding and interpretation of Christian scripture and doctrine (See Table 3). The majority of participants identified beliefs about sanctity and sacredness of all life as central to their views on abortion. They explained that people are created in God’s image, therefore human life has sacred worth which should be protected. The majority of participants stated views that public and pastoral care conversations about abortion should include recognition of the sacredness of life because Christian believers walk that experience (i.e. it is fundamental to Christian beliefs).

**Table 3:** Illustrative quotes of religious and moral belief sub-themes.

Common Sub-Themes	Illustrative Quote
Sacredness/Sanctity of Life	“I believe that all human beings are made in the image of God, that we all have a divine fingerprint on each of us and that leads me to want every life to be lived, right? Every aspect of the image of God to be lived out in this world because I believe in the sacredness of it.”
Fulfillment of potential	“This child has a destiny. You know we don't know what may come out of this situation. … as a pastor—I believe my thing [is] to get up with them as God biblically and theologically, which is this child here, something great can come out of something bad. Well, let's not say it's bad; let's not label it. Something great can come out of this.”
God’s forgiveness	“We don’t hold anybody in condemnation because you woke up one morning and did something crazy. God—there’s forgiveness for that. But we need to spread that abortion is not the way. It’s killing a mosquito with a candle. It’s just a baby. It’s another human life. It’s gonna be productive, hopefully, in somebody else’s life.”

Many participants across the attitude spectrum expressed the perception that abortion is ending life; however, participants had mixed views about when life begins and starts to bear the image of God. Participants with a “pro-life” attitude toward abortion described the beginning of life at early stages in fetal development, with some reporting life begins at conception, and others explaining that life begins when there is a fetal heartbeat. Participants expressing intermediate attitudes in the “gray area” did not have a consensus about when life begins, or, in other words, whether an abortion would end a life that was created in God’s image. One senior pastor with abortion attitudes in the “gray area” stated that he rejects the notion that “a fetus is just a grouping of cells.” Another senior pastor who also expressed attitudes falling in the intermediate “gray area” stated that he was not qualified to state when life begins but was confident it was not conception. He expressed uncertainty about when a fertilized egg starts “bearing the image of God,” but expressed that it was sometime between conception and birth. One participant with a “pro-choice” attitude toward abortion expressed beliefs about the beginning of life that were unique among other participants. She discussed views about life beginning when God’s spirits are brought into “our earthly journey” through the process of birth. She explained that any spirits that are not birthed go back to God and wait for the next chance at life, even as flesh dies during an abortion. She expressed this happens because God is loving:

*“If God is a God of love*, *why would God punitively respond to that entity of life that has no choice*? *I think God is bigger than that …”* (Regional Minister, Mainline Protestant)

#### Redemption

Many participants across the attitude spectrum expressed that there is a process of healing, redemption, or becoming “whole” that women must undergo following an abortion to resolve adverse psychological and spiritual effects. Most participants describe these effects, such as emotional guilt, regret, and spiritual effects, such as questioning whether God would forgive them. They expressed that these effects are often lasting and lifelong for those who cannot or will not “do the work [of] resolving their own minds.” Across abortion attitudes, participants expressed that adverse psychological effects are especially salient for women who never end up having children or learn later that they are infertile. In addition, some participants from both Mainline and Black Protestant churches expressed that there may be guilt, condemnation, criticism, and judgment from members of their congregation towards people who have an abortion. These participants expressed that stigmatizing responses from members of the congregation would continue to distress women after an abortion until they sought spiritual and emotional healing. Several participants explained the process of redemption and healing after having an abortion involves women reckoning with ending a life, recognizing they are covered by God’s grace and God does not condemn them, even if they may condemn themselves for their decision to terminate a pregnancy.

### Pastoral care

#### Across the abortion attitude spectrum

Many participants had little or no experience providing pastoral care related to abortion in their careers. They attributed their inexperience to perceptions that unplanned pregnancy and abortion not being major concerns in their churches and congregant perceptions that the church is not a safe place for conversations due to stigma. In addition, some men perceived women congregants would not come to them for pastoral care surrounding these issues or would prefer to go to women religious leaders. In such cases where leaders had little or no experience, they responded to hypothetical situations in which they would provide pastoral care to congregants considering abortion and often discussed these issues in the context of unmarried adolescents experiencing an unplanned pregnancy. Many participants expressed uncertainty about their qualifications to provide this type of pastoral care or lead faith-based health programs that included discussions of unintended pregnancy and abortion.

Across the spectrum of attitudes, participants expressed the importance of supporting a person facing an unplanned pregnancy and “journeying with” them in their decision-making, yet expressed a clear preference for continuing an unplanned pregnancy to term and utilizing adoption of the baby as a strategy (See Table 4). Paradoxically, all participants expressed their desire to counsel women away from abortion, or not “encourage” abortion as a solution to an unplanned pregnancy given beliefs about all life being sacred and needing protection. Several participants discussed the obligation to preserve God’s creation.

**Table 4:** Illustrative quotes for pastoral care sub-themes.

Common Sub-Themes	Illustrative Quote
“Well-informed decision”^1^	“I'm gonna pray with you that you're guided by the Lord, if you have that kind of faith. If you're a little weak in your faith, consider all the consequences that you can. Consider the impact on you, not just on the light that could be, but the impact on you. Don't ignore the fact that this will impact you. Don't think it's just gonna happen and you'll be fine, you'll move on.”
	“I would never encourage an abortion. I would always say that that is something if you're exploring or you're thinking about, please pray hard about it. I would, again, refer them to, first of all, a doctor, where they could get checked out and make sure that everything is healthy, but also give them the chance to get an ultrasound, hear a heartbeat.”
“Preserve God’s creation”^1^	“I think that the main role a religious leader can play is to assure the person that they are not going to have to be alone, they're not going to have to go through this alone, that there are resources to pull from, there are people that you can talk to, people you can rely on. There is information out there that can be handed on to you. I think, across the board, regardless of the churches, where they stand or where the pastor stands, I think that's the most important thing to do, is to say, "Hey, somebody is in your corner. Somebody is here with you." Then, from a theological standpoint, to me personally, I would highlight just the sacred worth that everybody has, that your life is important, the life of the unborn child is important. Every situation is different, but what we're trying to do here is preserve God's creation the best that we can in a very healthy way.”
	“I would first of all just tell them, ‘Let's really think about what honors God in this situation.’ If it's an issue of economics or whatnot, ‘hey, we'll be here as much as we can and deal with you. It's going to be tough. I'm not going to lie to you, it's going to be tough. But we're going to be here. This child has a destiny. You know we don't know what may come out of this situation.’ So I think that would be [the] thing as a pastor—I believe my thing [is] to get up with them as God biblically and theologically, which is this child here, something great can come out of something bad. Well, let's not say it's bad; let's not label it. Something great can come out of this.”
	please pray hard about it. I would, again, refer them to, first of all, a doctor, where they could get checked out and make sure that everything is healthy, but also give them the chance to get an ultrasound, hear a heartbeat.”
Support despite disapproval	“And I think what I said to her is that I, personally, did not subscribe to abortion. But my position does not hinder somebody else from making a decision for themselves. And so, whatever you do, I will support you. I think that where I wanted her to understand– and she asked, ‘What's your position?’ I'm going to be honest with, ‘This is what I believe but this is not based on what I, the ways on which God judges us. This is not God standard. This is from me and you must make your own decision. And in that, God is not an either/or God. God is not gonna condemn you to Hell but you have to be able to live with your decisions.’ And she eventually decided to abort. And from what I understand, had a happy life.” (Female Regional Minister, Mainline Protestant)
Instrumental Support	“We have responded to unplanned pregnancies by doing I think what we feel we're called to do, to make sure that the woman is getting health care, and provide the essentials for bringing a new baby–at least the physical part. You know, the crib, making sure they have–if they're not going to be able to breastfeed, that they have formula, blankets, all those things. And that's how we've responded in the past.”(Male Senior Pastor, Mainline Protestant)
“Love”^1^	"Well, Pastor's still going to love you just like he did yesterday, you know. That kind of thing. I'm not going to preach anybody into hell for an unplanned pregnancy. Life happens in so many other kinds of ways that anyone can be condemned for anything, and that's just not what I'm about. My thing is really telling someone their worth, you know, and making sure that they do those things that's proper. But when we fall, we fall, and God gives us an opportunity to get up again."(Male Senior Pastor, Black Protestant)
Encouragement to consider baby’s potential	“God can transform and change any situation, any circumstance. And, matter of fact, he specializes in that. … ‘So young lady, I understand you made a mistake. I get it. I'm not here to beat you up. I want to help you make wise decisions going forward. You don't know that that baby in your stomach won't be the next president of the United States … you don't know that that baby might not be the next doctor that discovers some cure for cancer. You don't know. But just because the child was conceived in these circumstances … doesn't mean that good can't come out of it.’ … listen, a bend in the road–which is what this is–is not a end in the road. We just gotta deal with it. Yogi Berra says, ‘What do you do when you come to a fork in the road? You pick it up.’ … In other words, you just deal with–and we just gonna keep going, you know?”

^1^ In-vivo codes

Similarly, many participants across the attitude spectrum emphasized the importance of expressing love when proving pastoral care for someone considering abortion, even if their theology led them to be morally opposed to abortion. All participants emphasized that they, the congregation, and God love a woman with an unplanned pregnancy that is considering abortion. Participants explained a loving response was a part of their duty as spiritual leaders. Participants with “pro-choice” attitudes and those falling in the “gray area” explained that part of their pastoral care would be to encourage their congregations to love and support someone considering abortion or who has had an abortion.

Several participants expressed that abortion will not separate women from the love of God, or the love of the participant, even if the decision to abort is not pleasing to God. Some participants equated abortion to sin such as divorce but explained that every sin, except blasphemy, is forgivable by God. These participants believed therefore, it was not their job to judge someone who has an abortion, even if they believe that abortion is ending a life. Some participants across the attitude spectrum expressed that God’s view of the sanctity of life is not punitive; therefore it is not appropriate for religious leaders to condemn women who have abortions to Hell or require them to publicly confess this sin. Additionally, many participants explained that people who have abortions are covered by God’s grace and forgiveness, religious beliefs that they would convey in their pastoral care.

Many participants across the spectrum of attitudes expressed the importance of not condemning a person because of abortion, citing scripture punitively, or passing judgment. A participant from an Episcopal church who identified as “pro-choice” said,

*“…I also think it’s letting them know that they’re loved*. *I go back to that*, *with–that’s–love will win*. *I know it’s become sort of a moniker and nobody takes it seriously*, *but– …It will*. *So how do we love*? *How do we love that woman who didn’t plan*, *and that baby that’s going to result from it*? *Or the woman that planned*, *and still she got pregnant*. *It’s not what do we with them*. *It’s how do we love them*, *and make sure they know they’re loved*? *That’s the part I see my role as*.*”* (Male Senior Pastor, Mainline Protestant)

When asked about how they would provide pastoral care for abortion, participants with “pro-life” and “gray area” attitudes cited examples of instrumental support they would provide as part of their pastoral care for unplanned pregnancies and emphasized providing support for continuing a pregnancy.

#### “Pro-life”

Participants with “pro-life” attitudes shared a belief that regardless of the intendedness of pregnancy, there are no “accidental children” because “God does not make mistakes.” They explained that they would draw upon this belief when providing pastoral care and would advise congregants considering abortion to first consider what God is calling them to do and consider the potential of their unborn child. Additionally, several participants with “pro-life” attitudes expressed that they would encourage people to first see an ultrasound or hear a fetal heartbeat before deciding on abortion. They cited examples of knowing people and hearing stories of young women who were seeking an abortion until they saw an ultrasound or heard a fetal heartbeat. While most participants across the attitude spectrum expressed they would encourage pregnant women to seek healthcare services, namely prenatal care, without being prompted, participants with “pro-life” attitudes discussed close ties to Crisis Pregnancy Centers and local pro-life advocacy groups they would call upon as resources. One senior pastor from a United Methodist Church explained his process in providing pastoral care to a young woman and making an appointment for her at a Crisis Pregnancy Center:

*“She didn't want to acknowledge that she was pregnant, so I set up with a crisis pregnancy center a time for her to go in for an ultrasound, start receiving prenatal care, and then she didn't show up … she didn't show up for the ultrasound, and all that. I was put in the awkward position of having to be a little bit aggressive with them, ‘Look, this is your life, and the child's life are at stake if you don't receive any prenatal care.’ … That was kind of a strange situation that I felt at some times like maybe I was overstepping my bounds by being pushy, but they weren't even talking to their parents about it. … That involved not just counseling but a lot of, I don't know what you would say, arm-twisting like, ‘You need to go get checked out, ’… It was a strange role, one of those they don't train you for in seminary, that's for sure. ”* (Male Senior Pastor, Mainline Protestant)

### Attitudes in the intermediate “gray area”

Participants with attitudes falling in the “gray area” explained they would encourage women to slow down and take stock of their available resources before making a decision about an unplanned pregnancy. These participants explained they would encourage congregants to consider if they had adequate child care, family support, and finances to parent a child. Some leaders expressed they would encourage women to careful gather all available information about options when faced with an unplanned pregnancy. These participants believed the congregation would likely encourage a fellow congregant with an unplanned pregnancy to keep the baby. Many suggested that they would counsel women considering abortion to not make a “rash” decision based on pressure from the congregation. In many of these cases, the participants expressed tension between beliefs shared in a pastoral care setting and beliefs of the congregation. These participants shared that they would encourage women to consider both options—to have an abortion or not have an abortion. Religious leaders with attitudes in the “gray area” expressed that they would advise a woman not to go through with an abortion unless there are where no other options. These leaders often emphasized contraceptive use as a primary method to prevent a pregnancy.

#### “Pro-choice”

Many participants who expressed “pro-choice” attitudes explained how they would provide support to a congregant seeking pastoral following an abortion. They expressed the process of helping a woman to reckon with ending a life and considering how God might view their action. They expressed women would need and want redemption, which would be available if sought. Others expressed they would attempt to use scripture to the best of their ability to encourage women seeking pastoral care, but would be careful not to “dictate what the bible says in terms of abortion” because little is written and free choice is allowed.

## Discussion

This study provides insight into the complex intersection of sociopolitical attitudes about abortion, religious and moral beliefs, and pastoral care among Mainline and Black Protestant religious leaders in Georgia. This analysis is part of a larger intervention development study to understand existing attitudes, norms, and values, in order to inform a faith-centered program on sexual and reproductive health that promotes compassionate attitudes and norms in Protestant religious contexts. While abortion attitudes fell on a spectrum ranging from “pro-life” to “pro-choice,” the majority of participants expressed attitudes intermediate, or in a “gray area,” between these views. Differences in abortion attitudes stemmed from varying beliefs on when life begins and circumstances in which abortion may be morally acceptable. Participants stressed that they would support women in pregnancy decision-making and advise them to make well-informed decisions irrespective of their own attitudes; yet, many described their lack of preparation and training to have these conversations.

Results showed numerous similarities and differences among Protestant religious leaders’ attitudes, beliefs, and pastoral care practices. For example, all participants agreed that they would participate in providing pastoral care for abortion at the initiation of congregants’ advice seeking. Other key similarities in pastoral care across the spectrum of abortion attitudes include recognition of the importance of the sanctity of life, emphasis on using scripture to encourage (i.e., not using scripture in a punitive manner), and acknowledgment that spiritual leaders are called to love and care for people unconditionally. In addition, many participants across denominations highlighted the psychological effects of abortion and a need for spiritual healing after abortion. Still, participants differed in their descriptions of their belief about when life begins, acknowledgement of moral agency to make pregnancy- and abortion-related decisions, and the circumstances in which abortion may be morally acceptable. For example, participants with “pro-choice” attitudes and participants with abortion attitudes in the “gray area” emphasized a preference against abortion but recognized a pregnant person’s moral agency to make decisions for their bodies and lives, whereas participants with “pro-life” attitudes did not express the same recognition.

These results demonstrate that Protestant religious leaders may provide pastoral care differently according to their abortion attitudes, thus varying their advice and recommendations; however, several misperceptions regarding abortion underlie religious leaders’ attitudes, beliefs, and pastoral care practices and run counter to existing scientific evidence. For example, many pastors described the adverse psychological effects of having an abortion including spiritual questioning, guilt, and lifelong emotional struggle and pain. In contrast, thirty years of research—including studies that measured mental and emotional distress before pregnancy—suggest legal induced abortion does not pose significant mental health risks for women [32–34]; however, evidence suggests being denied an abortion may result in negative psychological effects on women [35]. In addition, some religious leaders with “pro-choice” and “gray area” attitudes stated they would advise against the use of abortion as contraception despite clinical guidelines clearly distinguishing between contraception as a form of primary pregnancy prevention while abortion is a form of pregnancy termination [36]. Further, despite religious leaders beliefs that women use abortion “conveniently” or as a primary pregnancy prevention method in the United States, people have abortions for diverse and interrelated reasons (e.g., 73% cited inability to afford a child and 74% cited having a baby would interfere with work, education, or ability to care for dependents) [37] and over half of U.S. abortion patients were using a contraceptive method when they became pregnant [38].

Participants who identified a “pro-life” attitude towards abortion cited informational support that encouraged women to continue their pregnancies and carefully consider the potential of the unborn child. Many of these participants expressed that their pastoral care would include instrumental support in the form of making referrals to Crisis Pregnancy Centers (CPCs) and pro-life advocacy organizations. Those with a “pro-life” attitude explained that CPCs were reliable sources of information and would help women to get prenatal care. They explained that women could go to a CPC to see an ultrasound or hear a fetal heartbeat. Some participants with a “pro-life” attitude explained that they would advise women to seek services at a CPC before deciding to have an abortion. Protestant religious leaders may view CPCs as reliable healthcare services because of their emphasis on religious ideology; however, evidence suggests women seeking care at these clinics “do not receive comprehensive, accurate, evidence-based clinical information about all available options” [39].

Only participants with “gray area” or “pro-choice” attitudes expressed a duty to confront stigmatizing attitudes and behaviors towards women who experience abortion. These participants discussed obligations to encourage empathy and dispel stigmatizing attitudes and treatment within their congregations. While these participants were the only religious leaders who expressed these views, all participants emphasized the importance of empathy, love, and compassion for others. Thus, Protestant religious leaders may be key players in confronting abortion stigma in Mainline and Black Protestant churches and should be involved at the onset of efforts to destigmatize abortion and “shift the cultural conversation from one of judgment to one of empathy, compassion, and affirmation of people’s moral agency” [17]. These findings hold promise for informing the development of multi-level faith-based interventions and secular and faith-based partnerships to reduce abortion stigma.

Many religious leaders cited that they had not had formal training on providing pastoral care for any sexual and reproductive issue, let alone abortion. Pastoral care training and interventions should be developed that emphasize Christian beliefs and value the sanctity of life and integrate public health recommendations. Key intervention components should: include information on evidence-based healthcare services and local supports, address beliefs about both the psychological and the spiritual effects of abortion, dispel misinformation, and integrate strategies to reduce abortion stigma. Some religious scholars are already considering faith-based, reproductive justice, and moral arguments for supporting abortion [19, 40–44]. Existing moral arguments for legal abortion and this qualitative evidence could be used to inform intervention development.

These findings should be interpreted in context of the limitations and strengths of the study. Our diverse sample of Mainline and Black Protestant churches represent an array of perspectives on abortion and pastoral care, but cannot (and were never intended to) be generalized to all religious denominations (e.g., Evangelical Protestant, Catholic, and non-Christian groups were purposefully excluded). Additionally, only few women were recruited in this study, which somewhat reflects the gender makeup of senior leadership positions in Protestant religious institutions which are male dominated. It is possible that women lay leaders are providing pastoral care to congregants as well and may have different insights on the provision of pastoral care for abortion. Future research should seek to specifically recruit Mainline and Black Protestant women religious leaders. Investigation of how these findings compare to abortion attitudes and pastoral care practices among other religious traditions in Georgia, the Southeast region, and other states is warranted. It is possible the participants included in this study represent a group with more liberal views on abortion given the fairly liberal stances of their denominations compared to other religious traditions. It is a strength of this study, however, that many diverse political and socio-political attitudes were observed among participants from the same denominations.

## Conclusion

The insights provided by this study help provide an understanding of how Mainline and Black Protestant religious leaders in Georgia provide pastoral care to congregants regarding decision-making for unintended pregnancies, including abortion decisions. Protestant religious leaders may play an important role in providing social support, and facilitating access to information and healthcare services. Finally, these findings help to understand complex religious and cultural perspectives on abortion and how these attitudes influence pastoral care. This is an important step towards creating partnerships between public health and Protestant religious organizations that improve reproductive health outcomes, reduce abortion stigma, and respect the intrinsic value of religious traditions on their own terms. Future research on the larger intervention development project will take another step by collecting congregant’s perspectives on pastoral care for unintended pregnancies and abortion, and investigate the barriers and facilitators to receiving support from their religious communities.

## Supporting information

S1 Appendix Interview guide. (DOCX)

S2 Appendix Codebook. (DOCX)
