# Supplementary material for: Abortion attitudes, religious and moral beliefs, and pastoral care among Protestant religious leaders in Georgia
Source: PLoS One. 2020 Jul 17;15(7):e0235971. doi: 10.1371/journal.pone.0235971 (PMC7367465; doi:10.1371/journal.pone.0235971)
Supplement: S1 Appendix — (DOCX) [file pone.0235971.s001.docx]

**S1 Appendix. Interview Guide**

1. **Warm-Up**

*I would like to begin by asking you a little bit about your role in your church community.*

1. What are your daily responsibilities in your church?

*Probe:* What responsibilities do you have within your church community related to Sexual and

reproductive health (family planning, sex, pregnancy, and birth control?)

1. **Sexual and Reproductive Health**

*Next, I would like to ask about sexual and reproductive health issues in your church community.*

1. What are the sexual and reproductive health issues affecting people in your church community?

*Probe:* For men? For women? For young people?

1. How do conversations about sexual and reproductive health happen in your church community?

*Probe:* Your role in these conversations?

1. **Sexual Activity & Unplanned Pregnancy**

*Next, I would like to understand views on sexual activity and unplanned pregnancy.*

1. As a religious leader, what are your views on sexual activity?

***Probe: For men? For women? For younger people? Other groups of people?***

1. How do your views on sexual activity compare to your church community’s views?

*Probe:* Other Leaders/Congregants/Denomination

1. What happens to someone as a result of an unplanned pregnancy?

*Probe:* spiritual effects, effects on relationship with their church community, economic effects, health effects,

1. Can you describe a time you provided pastoral care to someone for an unplanned pregnancy?

*Probe:* What did you say? Advice/recommendations? What did you do? What resources did you rely on? If no experience, how would you provide pastoral care?

**C. Abortion**

*Now, I would like to understand views on abortion.*

1. What are your personal views on abortion?

*Probe:* Moral acceptability? Moral autonomy? Legality?

1. What happens to someone as a result of having an abortion?

*Probe:* spiritual, effects on relationship with their church community, health effects

1. What has shaped your views on abortion?
2. How do your current views on abortion compare to your views on abortion at the beginning of your career?

*Probe:* Causes of change?

1. How do your views on abortion compare to your church community’s views?
2. Can you describe a time you provided pastoral care to someone considering abortion?

*Probe:* What did you say? Advice/recommendations? What did you do? What resources did

you rely on? If no experience, how would you provide pastoral care?

1. Can you describe a time you provided pastoral care to someone after having an abortion?

*Probe:* What did you say? Advice/recommendations? What did you do? What resources did

you rely on? If no experience, how would you provide pastoral care?

**E. Perspectives on Faith-Based Strategies on Addressing SRH**

*Next, I’d like to get your suggestions on the best strategies for addressing sexual and reproductive health concerns.*

1. How important are programs and services that discuss sexual and reproductive health for your church community?

*Probe:* How important are programs focused on [sexual activity/unplanned pregnancy/ abortion]? Why?; [If important]: What messages would they cover?

1. How appropriate are programs and services that discuss sexual and reproductive health for church settings?

*Probe:* How appropriate are programs focused on [sexual activity/unplanned pregnancy/ abortion]? Why? What messages should these programs and services deliver?

1. What is the best way for your church community to receive support on sexual and reproductive health?

*Probe:* Decisions around pregnancy?

1. If you were in charge of developing a program for Georgia’s Protestant churches related to sexual and reproductive health, what would this program look like?

*Probe:* What topics would it cover? What messages are important? What are some challenges to implementing such a program?

**F. Wrap-Up**

*We are coming to the close of the interview now, I have two last questions.*

1. What advice would you give the research team about working with churches to address congregants’ sexual and reproductive health concerns?
2. What other thoughts would you like to share related to what we discussed?

***That completes the interview. Thank you for taking the time to share your thoughts with me!***
