# Supplementary material for: Abortion attitudes, religious and moral beliefs, and pastoral care among Protestant religious leaders in Georgia
Source: PLoS One. 2020 Jul 17;15(7):e0235971. doi: 10.1371/journal.pone.0235971 (PMC7367465; doi:10.1371/journal.pone.0235971)
Supplement: S2 Appendix — (DOCX) [file pone.0235971.s002.docx]

**S2 Appendix. Codebook**

Key: **CODENAME** (in Dedoose) – definition

Parent codes are justified to the left and sub-codes are bulleted

**ABORTION**  - Use for any mention of induced abortion, or the deliberate termination of a pregnancy.

**ADOPTION/FOSTER CARE** – Use for any mention of adoption or foster care, or the placement of a child in another person’s care, whether formal or informal, short or long-term care.

**ATTITUDES AND BELIEFS** - Use this code for any mention of religious leaders and/or congregant's beliefs and attitudes (positive or negative evaluation of beliefs, and strength of that belief) about health topics

- **DISCORDANT VIEWS** - Any discussion of discordant attitudes and beliefs between the religious leader and their church, congregants, other leaders, community, or other institutions. For example, this code includes mention of discordant views between the religious leader and teachings they received during seminary.
- **SHIFT IN ATTITUDES & BELIEFS** - Includes discussion of how current beliefs and attitudes compare to an earlier point in the participant's life. Also includes mention of beliefs and attitudes that have not shifted over time. This code does not include shifts in congregant- or church-level beliefs or attitudes, only the participant's personal views.

**AUTONOMY** – Any discussion of the ability to make independent, non-coerced decisions; Self-determination (the ability to determine one’s own fate/destiny/goal, including the behavioral path to that fate/destiny/goal); This might also include mention of self-efficacy (belief in one’s ability to enact a specific behavior) or agency (the means to act or exert power); This extends beyond reproductive behaviors or decisions.

- **CHOICE** - References or any mention of "choice" or a "woman's choice" within any pregnancy decision making (e.g parenting, abortion, adoption, etc.) This code also includes any mention of the process to choose an abortion and references to the cognitive processes in weighing options. For example, includes discussion of induced abortion (double code with "abortion."). Double-code when there are references to both pregnancy decision-making and other domains of autonomy (e.g., financial autonomy).

**BEST WAYS FOR SUPPORT** - Use this code for any mention of the best ways for congregants to receive support related health issues. This code aligns with, but is not exclusive to responses to Question #14 on the interview guide. "What is the best way for your congregation to receive support in issues related to family planning, sex, pregnancy, and birth control concerns?

**CONGREGATION** - Includes references to the religious leader's congregation or congregants, the people who attend and participate in church services and activities

**DISABILITY/SPECIAL NEEDS** - Any mention of fetal anomalies or children with born with disabilities and birth defects. Double code with abortion and choice when referring to a(n) (un)justifiable reason for abortion.

**DISCLOSURE** - Use for reasons why people chose to or chose not to disclose their health issues or concerns to their church. Includes discussion of disclosure (or choosing not to disclose to the church or religious leader(s)) having or considering an abortion

**DOCTRINE** - Any mention of beliefs or a set of beliefs held and taught by a Church that are generally accepted by its members. Examples include omnipotence (i.e. God is all-powerful with unlimited ability) and God’s purpose.

- **LIFE** - Any mention of the phrase "sanctity of life." Also includes discussion of the sacredness of life and people being "made in God's image." Consider double coding with abortion if discussion includes beliefs about when life begins or discussions about ending a life (by abortion; "killing"). Includes general mention of when life begins
- **REDEMPTION** –Any mention of redemption within narrative, this includes personal redemption (e.g. after an abortion), social redemption (e.g. a women can be welcomed back into the church), spiritual redemption, redemption on the part of the fetus (e.g. a child did not choose to be born in those circumstances). Double-code with abortion, marriage, pregnancy, etc. as appropriate
- **RELIGIOUS ACCOUNTABILITY** - Any reference to being held accountable or upholding accountability for the way scripture is being interpreted or used; the sanctioned use of scripture to increase/decrease/maintain pastor or Christian credibility; justification for policing/maintenance/creation of boundaries within the church, with an understanding of the tenets of scripture or doctrine
- **SCRIPTURE** - Use for specific mention of scripture, or the sacred writings in the bible, and religious text. Includes scripture religious leaders use when providing pastoral care. Also use this code for mentions of "The Gospel" or the "Word of God," and when scripture is directly paraphrased.

**GOD** - Includes mention any of God, "The Lord," or the "Father in Heaven." Also includes external attribution around a higher power and mention of God’s authority. For example, use this code for mention of the "Grace of God."

**FAITH AND SPIRITUALITY** – Use this code for mention or description of an individual’s search for and process of searching for transcendence, meaning, purpose and/or significance.

**GENDER** - Includes any mention of gender identity, gender norms, or gender roles. Also includes specific mentions of sexism.

- **MAN’S ROLE** – Any reference to the role of a man in society, including in sexual and reproductive experiences and decisions (sexual behavior, pregnancy decision-making, parenting, adoption etc.). This code includes (but is not limited to) mentions of support around pregnancy decision-making, providing counsel around pregnancy decision-making, and the role of the father or partner of a pregnant woman (e.g. “when there is not a lot of value placed on the dad. And he’s the missing key.”)
- **WOMAN’S ROLE** – Any mention of the role of a woman in society, including in sexual and reproductive experiences and decisions. This code includes (but is not limited to) any reference to the role of a woman in pregnancy decision-making, support around pregnancy decision-making, providing counsel. Includes specific mention of gender.

**GREAT QUOTES** - Use this code for compelling excerpts (i.e. juicy quotes :)

**HEALTHCARE SERVICES** - Any mention of the medical professionals, organizations (e.g. abortion clinics), and ancillary health care workers who provide medical care to those in need. Also use this code for mentions of specific types of healthcare service such as ultrasound imaging or prenatal care.

- **CRISIS PREGNANCY** – Any discussion of Crisis Pregnancy Centers. Also use this code for specific mention of Crisis Pregnancy Ministries.

**LIFE ASPIRATIONS** - Any mention of the pregnant person's life aspirations, attainment of expected goals, or achievements. Examples: mentions of desires to finish high school or college, start a business, get married, being a parent, how many children desired, etc.

**LOVE** - Use this code for any direct mention of love. This code includes mention of God's love or congregant's loving one another (congregant response). Also use this code for mentions of "agape," "phileo," and "the eros."

**MARRIAGE** - Use this code for any mention of marriage or divorce. Examples include discussion of marriage counseling or pre-marital sex. This code includes references to the leader's own marriage or spouse.

**MORALITY** - Use this code for discussion of moral acceptability/unacceptability. For example, this code includes discussion of circumstances that make abortion morally acceptable and also circumstances that make abortion morally unacceptable (e.g. rape and incest as acceptable reasons for induced abortion and induced abortion to make the mother's life more convenient as an unacceptable reason.)

- **SIN** - This code includes any direct mention of sin. Transgression or wrongful act against a law of God/the divine law.

**OTHER HEALTH** - Use this code for any mention of health topics not directly related to sexual and reproductive health. Examples include mention of mental health (unless it is a specific psychosocial effect of a reproductive experience or event - in this case, code “psychosocial effects”), cancer (excluding reproductive cancers), or alcoholism/substance abuse.

**OTHER SRH** - This code includes mention of other sexual and reproductive health topics, not including induced abortion, unintended pregnancy, and sex & sexuality. Examples include mention of menopause, contraceptives, family planning, HIV and other STIs; Also includes mention of spontaneous abortion (i.e. miscarriage), mentions of reproductive cancers (breast, ovarian, cervical etc.), birth and sexual assault.

- **FERTILITY** – Any mention of the biological capacity to be fertile or the number of children that an individual has over their life course. Double-code with “Life Aspirations” as appropriate.

**PARENTING** - Any mention to the process or act of promoting and supporting the physical, social, emotional, intellectual development from infancy to adulthood

- **COST OF PARENTING** – any reference to the financial, social, and emotional costs of promoting child development

**PASTORAL CARE** - Used when a participant discusses providing pastoral care to others. Pastoral care includes acts, such providing emotional and/or spiritual guidance, support, and advice, directed toward the "healing, sustaining, guiding, reconciling, and nurturing of persons." Examples include visitation, counseling, and "ministries of shared presence, listening, and support," and praying with someone in crisis. This code does not include delivery of sermons.

- **BARRIERS** - Use for discussion of barriers to providing pastoral care. For example, culture of distrust, people not talking about particular issues with their pastor, or pastors not wanting to provide emotional, spiritual or other support around a particular topic (in the context of pastoral care). Double-code with pastoral care team, qualifications & training and disclosure where appropriate.
- **FACILITATORS** - Any mention of factors that facilitate provision of pastoral care. For example, when a pastor shares their own experiences and stories (double-code with personal experiences), is able to relate to their congregants (related to being close in age or sharing the same gender), and has spent a longer length of time in their position or at that church. Double-code with pastoral care team and qualifications & training where appropriate.
- **PASTORAL CARE TEAM** - Use this code for mentions of people in the church that religious leaders and/or congregants rely on for support or avoid when providing or receiving pastoral care.
- **QUALIFICATIONS & TRAINING** - Use for any discussion of the religious leader's self-perceived qualifications (or lack thereof) to provide pastoral care. This code includes any mention of actual training religious leaders have received whether in seminary or elsewhere. Also includes any mention of qualifications and training regarding providing pastoral counseling.

**PERSONAL EXPERIENCES** – Any narrative or story of interviewee or other close friends, family members and acquaintances that is linked to spiritual beliefs and/or sexual and reproductive health values (beliefs/attitudes). Also includes direct mention or discussion of not having a specific experience in one’s personal life or in their role/interactions at church (e.g. mention of not having experience providing pastoral care for unplanned pregnancy)

**POLICY/LEGALITY** - Any mention of policy and legislation, the process of policymaking, and attitudes about policy or legislation regarding health topics. Also includes mention of the present political climate, activism, or protesting.

**POLITICAL IDENTITY** - Includes any mention of the participant's or others’ (incl. congregants') political affiliation. For example, use for specific mention of party affiliation, and mention of conservative, liberal, moderate political views or identity.

**PREGNANCY** - Use this code for any mention of pregnancy

- **UNINTENDED/AMBIVALENT PREGNANCY** - Use for any mention of a pregnancy that is (perceived to be) mistimed, unplanned, or unwanted. This code includes any mention of lack of clarity surrounding pregnancy intentions (i.e. ambivalence). For example, use this code when participant mentions their or someone else’s uncertainty about wanting to be pregnant or when they want to become pregnant.

**PRIORITIES** - This code includes any discussion of the health priorities and biggest concerns of congregants. Also includes mention of the religious leader's health priorities, biggest concerns, and any mention of the congregation’s pastoral care or supportive care needs relating to sexual and reproductive health. This code aligns with, but is not exclusive to question #3 on the interview guide, "think about the people in your church, what are their biggest concerns involving reproductive health?"

**PROGRAMS** – Any mention from the participant of the program, project or research. Excludes child codes.

- **ADVICE TO ENFAITH** - This code includes discussion or mention of any advice about working with churches to address health concerns. Aligns with, but is not exclusive to question #16 on the interview guide, "what advice would you give the research team about working with churches to address congregants' reproductive health concerns."
- **APPROPRIATENESS OF PROGRAMS** - This code includes any discussion of the appropriateness or acceptability of health programs and services delivered in church settings. Aligns with, but is not exclusive to responses to question #7 on the interview guide, "How appropriate are programs and services that discuss the topic of unplanned pregnancy for church settings?" Also aligns with, but is not exclusive to question #13 on the interview guide, "How appropriate are programs and services that discuss the topic of abortion for church settings?"
- **PROGRAM SUGGESTIONS** - Includes any discussion of suggestions for faith-based reproductive health programming. This code aligns with, but is not exclusive to question #15 on the interview guide, "if you were in charge of developing a program for Georgia's Protestant churches related to reproductive health, what would this program look like?"

**PSYCHOSOCIAL EFFECTS** - Any mention of the individual, intra- and inter-personal psychological effects of reproductive experiences (pregnancy, pregnancy decision making – to include parenting, adoption, and abortion, miscarriage, infertility). This code also includes mention of guilt, remorse, regret, fear, and emotional consequences between people when discussing reproductive experiences. For example, double code with "abortion" for discussion of short-term and long-term emotional consequences of induced abortion.

**RACE** - Includes references to someone's or a group of people's racial identity

- **THE BLACK CHURCH** - Includes mention of Black Protestant churches or "The African American Church." Also use this code for mentions of the "Black Preacher" or African-American pastors/preachers.

**RESOURCES & REFERRALS** - Use for mention of community and church resources that religious leaders do or would rely on when providing pastoral care. Includes references to utilizing the human resources in the church (e.g. doctors or nurses in the church). For example: advising a pregnant person to see the church minister who is a licensed professional counselor

- **OUTSIDE INSTITUTIONS**  - Includes mention of outside institutions as resources for the church or for congregants (e.g. The Centers for Disease Control, CDC, or the Health Department.) Also includes mention of connections and collaborations with other churches and/or denominations.

**SEXUALITY** - For use with any mention of sexual knowledge, beliefs, attitudes, and values. Applies to social attitudes in addition to individual attitudes (e.g. the church's stance)

- **LGBTQ**  - This code encompasses both (1) romantic, emotional, or sexual attractions toward other people of the same sex or gender, or toward people of more than one gender; and (2) sense of self as a sexual being, including transgender, gender variant, or gender non-conforming identity. This also includes the church's stance/change on same sex marriage or homosexuality
- **PORNOGRAPHY** – Any mention of pornography
- **SEXUAL BEHAVIOR** - References to interactions of a sexual nature. For example, lust, sexual exploration, and sexual touching. Includes explanation of relationships that lead to sex, discussion of abstinence, and discussion of virginity.

**SOCIAL IDENTITY** - Use this code for any mention of the participant's or congregants' social identity. For example, use for specific mention of pro-life or pro-choice identity; feminist identity; religious identity.

**SOCIAL INCLUSION** - Any mention of rejection, exclusion, and social distancing or any mention of social inclusion and cohesion. For example, discussion of leaving the church, being ostracized, or being welcomed in to the church

**SOCIAL NORMS** – The customary rules of behavior that govern our interactions with others. They are “common standards within a social group regarding socially acceptable or appropriate behavior in particular social situations, the breach of which has social consequences. Any mention of descriptive norms (the norm that indicates the way that most people act) and injunctive social norms (the type of norm that others approve of in a given situation within the larger community (ex. Macon, Georgia, the U.S.). Can also be applied in discussion of the degree to which people comply with social norms. For example, apply to discussion of how young people act generally, given society patterns of behavior (coded as social norms, young people, and sexual behavior).

- **CHURCH NORMS** - Social behavior of church or church members that is accepted as normal and/or that individuals are expected to conform to. Also includes informal rules that govern behavior of people affiliated with a church.
- **FAMILY NORMS** – Social behavior of families, a family unit or family members that is accepted as normal and/or that individuals are expected to conform to. Also includes informal rules that govern behavior of people affiliated with a family. For example, could be used for description of the norm that most families don’t eat dinner together, and the approved behavior would be for most families to eat dinner together.

**SOCIAL SUPPORT** - Use this code for any mention of social support. This includes instrumental support (e.g. giving money, food), informational support (e.g. advice), and emotional support (e.g. shoulder to cry on). For example, use this code for references to community or pastoral support that do not include counseling and prayer; references to hospital visits, child care, and/or monetary support, and affirmation of choices.

**WHOLENESS** - The state of being perfectly well in body, soul (mind, will and emotions) and spirit. Complete sanctification and restoration. Includes references to caring for the whole person or to the wholeness of people. A reference to completeness or incompleteness of a person in spiritual terms.

**YOUNG PEOPLE** - Any mention of young people between the ages of 12 to 19, adolescents, teenagers, young people, or young adults. For example, use this code (along with the relevant "pregnancy" code) for discussion of teenage pregnancy.
